# Microbial production of butyl butyrate: from single strain to cognate consortium

**DOI:** 10.1186/s40643-021-00403-4

**Published:** 2021-06-12

**Authors:** Jean Paul Sinumvayo, Yin Li, Yanping Zhang

**Affiliations:** 1 CAS Key Laboratory of Microbial Physiological and Metabolic Engineering, State Key Laboratory of Microbial Resources, Institute of Microbiology, Chinese Academy of Sciences, Beijing, 100101 China; 2 University of Chinese Academy of Sciences, Beijing, 100049 China

**Keywords:** Microbial synthesis, Butyl butyrate biosynthesis, Metabolic engineering, Cognate consortium

## Abstract

Butyl butyrate (BB) is an important chemical with versatile applications in beverage, food and cosmetics industries. Since chemical synthesis of BB may cause adverse impacts on the environment, biotechnology is an emerging alternative approach for microbial esters biosynthesis. BB can be synthesized by using a single *Clostridium* strain natively producing butanol or butyrate, with exogenously supplemented butyrate or butanol, in the presence of lipase. Recently, *E. coli* strains have been engineered to produce BB, but the titer and yield remained very low. This review highlighted a new trend of developing cognate microbial consortium for BB production and associated challenges, and end up with new prospects for further improvement for microbial BB biosynthesis.

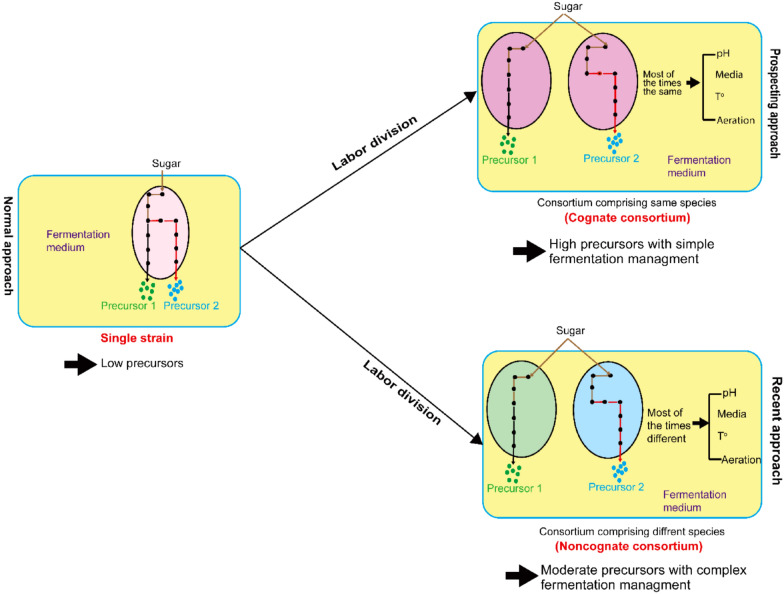

## Introduction

Butyl butyrate (BB) is an ester compound, which has been broadly exploited in the food, cosmetic, detergent, chemical, and pharmaceutical industries (Brault et al. 2014). Various food beverages contain added BB to enhance their taste, and the fragrance cosmetic industries add BB to achieve a particular fruity or floral scent (Iwasaki et al. 2012). Furthermore, BB is also used as a solvent in the plastic, texture, and fiber industries, and it is also an important extractant in the processing of petroleum products. Apart from a unique fruity odor that attributes BB to versatile applications, the octane rating (97.3) of BB is higher than the minimum standard rating (95) required in EN 228 (European Standards for Gasoline). Besides, BB, with a melting point below − 47 °C and a flash point above 38 °C, is compatible with kerosene and also showed miscibility with kerosene at low temperatures. With such excellent compatibility with, and similar properties to, gasoline, jet fuel, and diesel components, BB is considered as a potential aviation fuel constituent which makes it one of the mostly needed esters (Rhodri et al. 2013; Xin et al. 2019).

Generally, short-chain fatty acid esters, including BB, will have an estimated global market exceeding the US $ 2 billion by 2022 (Xin et al. 2018). BB can be produced by chemical synthesis or extracted from natural sources (Longo and Sanromán 2006). Chemical synthesis involved the acid-catalyzed esterification of butyrate serving as donor with butanol acting as acceptor. However, this process has often encountered the challenge of hydrolysis at ambient temperature (Δ*G* = − 5 kcal/mol) in the presence of water (Liu et al. 2006; Nelson and Cox 2008). In addition, chemical synthesis of BB may be considered as less attractive and unsustainable process due to the use of harsh conditions involving high concentrated sulfuric acid, toxic solvent and high-energy conversions, which sometimes result in environmental damage as well as carbon footprint.

Besides, chemical approach also suffers from the lack of substrate selectivity, creating racemic mixtures with undesired side products that reduce synthesis efficiency and increase production costs (Vandamme and Soetaert 2002). Some examples have been given showing ester synthesis through enzymatic catalysis. Here, butyrate serving as donor and butanol acting as acceptor are all externally added to the system (van den Berg et al. 2013; Abbas and Comeau 2003; Martins et al. 2013; Lorenzoni et al. 2012; Santos and de Castro 2006). In parallel to the chemical synthesis and enzymatic catalysis methods of BB production, food raw materials such as fruits have been used to extract BB. However, given that people need fruits to gain healthy nutrients, the high requirements of fruits to produce BB will cause food shortage, which may affect human nutrients satisfaction. Although, the production of fruits grew significantly and they are used as raw materials for BB and other short-chain esters extraction, this method resulted in low production of BB (Pinheiro et al. 2008; Miguel Espino-Díaz et al. 2016).

It is therefore important to seek an alternative approach, i.e., fermentation, for environmentally friendly and sustainable production of BB. This approach can be beneficial over extraction method from fruits since it precludes high utilization of raw materials, which compete with human need. One of the advantages of the fermentation-based approach, apart from its environmental friendliness, is the high selectivity and the relatively low cost of the final product (BB). However, many works are needed to engineer amenable microorganisms since most of BB producer strains do not have relative native pathways capable to generate main precursors.

**Fig. 1:**
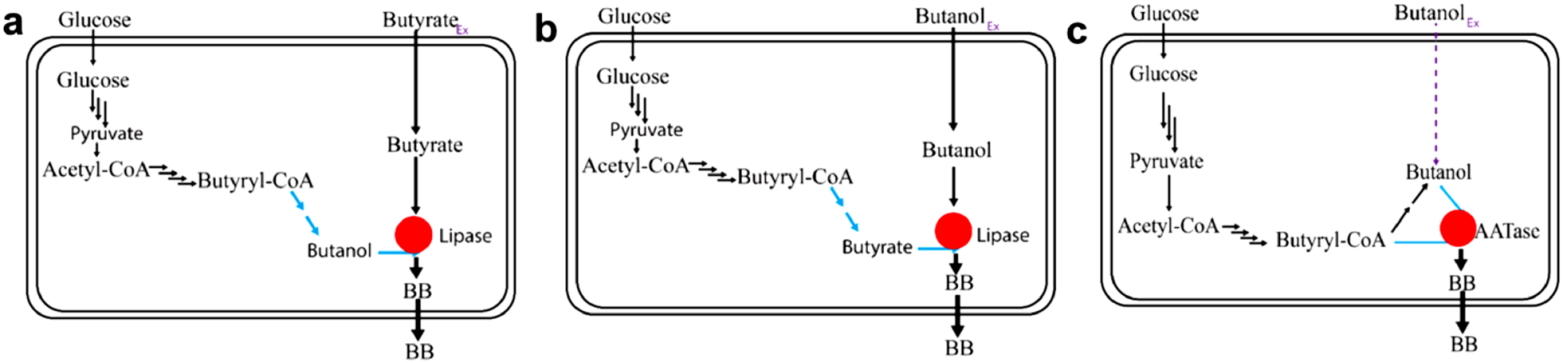
Possible strategies to produce BB via a single strain aided by lipase or alcohol acyltransferases (AATs). **a** The microorganism can produce butanol then external butyrate and lipase are added in the fermentation to undergo the esterification process. **b** Some *clostridia* strains, such as *C. tyrobutyricum,* can produce high butyrate. Thus, it requires an external addition of butanol and lipase to the fermentation for butyl butyrate synthesis. **c** Engineered microorganisms with the strong C4 pathway expressed with AAT can accumulate butyryl-CoA and butanol for BB biosynthesis. In case they have low levels of aldehyde and alcohol dehydrogenase activity, butanol needs to be externally supplemented to the fermentation medium (indicated by a purple dashed line)

To date, there are two possible biological approaches to produce BB through single microbial strain (Fig. 1). The first uses bioconversion of one precursor biologically produced and the other one externally added to the fermentation medium (Fig. 1a, b), and the second uses engineered microorganisms to produce BB de novo from glucose by fermentation, but butanol can also be added in case enzymes converting butyryl-CoA to butanol have low activity (Fig. 1c).

*Clostridia* species have been widely used as single strain for BB production. For example, *C. acetobutylicum* that is widely used in ABE (acetone–butanol–ethanol) fermentation has the pathway for production of butyrate and butanol. It produces butyrate in the acidogenesis phase, while butanol is produced in the solventogenesis phase, at the expense of butyrate. Thus, butyrate needs to be added to the fermentation broth of *C. acetobutylicum* to produce BB (van den Berg et al. 2013; Xin et al. 2016). *C. tyrobutyricum* can naturally produce butyrate, but it lacks the pathway for butanol production (Zhang et al. 2017). Consequently, butanol needs to be added to the fermentation broth of *C. tyrobutyricum* to produce BB.

*E. coli* has also been deployed as a host for BB production (Baek et al. 2013; Bill 2014; Pontrelli et al. 2018). This species naturally does not have related pathways for either butanol or butyrate production. Therefore, heterologous pathways for butyryl-CoA/butanol are required. Recently, Kruis et al. (2019) has intensively reviewed microbial production of esters include BB by using single strains, with particular focus on the exploitation of alcohol acyltransferases (AATases). Since BB is produced from two butanol and butyrate, one alternative approach is to design a microbial consortium and divide the labor into two strains. Such a consortium might be able to simultaneously produce butanol and butyrate in a given ratio, and achieve de novo biosynthesis of BB from glucose. Thus, in this review, taking BB as an example of ester compounds, we highlight the advances on its production through single native microorganisms, metabolically engineered strains, and microbial consortium approaches. Finally, future challenges and proposing prospects for future studies on microbial BB biosynthesis were proposed and discussed.

## *Clostridia* species as microbial cell factories for BB biosynthesis

Many *Clostridia* species can produce butyrate and/or butanol from butyryl-CoA, which make them promising microbial cell factories for the production of BB. Hence, different strategies have been proposed for microbial ester synthesis in *Clostridia* species (Fig. 1). Naturally, *Clostridia* strains, respectively, generate acids and alcohols during acidogenic and solventogenic phases. In this process, butyrate and butanol are the two main products in the fermentation broth with a constantly changing ratio, due to conversion of butyrate into butanol, which lead to low BB production (Xin et al. 2019). To date, there are no clear practical guidelines for the required concentration of substrates for ester synthesis. In theory, high butyrate concentration in the reaction system may drive the equilibrium of the reaction to the BB side. However, it was also reported that high butyrate concentration inactivated the lipase enzyme probably due to very low pH (< 3) associated with such high acid concentration, which led to poor esterification (Devi et al. 2017). Therefore, either butanol or butyrate should be supplemented in the system based on which substrate is in the shortage state (Fig. 1a, b).

Very recently, much attention has been paid to produce BB by feeding the exogenous precursor(s) (Table 1). However, this strategy may complicate the fermentation process by constantly controlling substrates levels required to enable the esterification reaction. A recent study, focusing on BB production by in situ esterification and extractive fermentation carried out in *C. tyrobutyricum* indicated that to achieve 34.7 g/L BB, butanol must be maintained at 10 g/L until the completion of the fermentation (Zhang et al. 2017). Seo et al. (2017) used a similar strategy to produce BB by *C. beijerinckii*. To this approach, a titer of 3.32 g/L BB was achieved in the hexadecane phase when supplementing 5 g/L butanol as a required precursor. van den Berg et al. (2013) used fed-batch fermentation of *C. acetobutylicum* by feeding glucose and butyrate (unstated quantity) which resulted in 5 g/L BB in the hexadecane phase. *Clostridium* sp. strain BOH3 was able to ferment xylose to produce 22.4 g/L BB in a fed-batch fermentation. It requires addition of 7.9 g/L butyrate to support BB production as this strain was not able to produce sufficient butyrate. Yet, it is noted that the cost of substrates themselves can jeopardize the economic viability of the process (Xin et al. 2019). Therefore, it is important to develop a straightforward process that is not dependent on the precursor(s) supplementation for BB biosynthesis.

**Table 1:** Comparison of recent studies for microbial ester biosynthesis by *Clostridia* species

Strain	Strategy	Exogenous precursors	Titer	References
*C. tyrobutyricum*	Fermentation of 50 g/L glucose to butyrate, then add 5.0 g/L lipase with hexadecane as extractant	Keeping butanol at 10.0 g/L during fermentation	34.7 g/L BB	Zhang et al. (2017)
*C. beijerinckii* spo0A mutant	Fermentation of glucose to butyrate and butanol, then feed lipase with hexadecane as extractant	5 g/L butanol was added in a continuous agitation	3.32 g/L BB	Seo et al. (2017)
*C. acetobutylicum*	Fed-batch fermentation by feeding glucose and butyrate, 2500 U (3.31 g) lipase was added with hexadecane as extractant in 1.5 L culture medium	Unstated quantity	5 g/L BB	van den Berg et al. (2013)
*Clostridium* sp. strain BOH3	Fed-batch fermentation of xylose using both in vivo and exogenous lipases, and kerosene as extractant	7.9 g/L butyrate was added to the fermentation	22.4 g/L BB	Xin et al. (2016)
*C. acetobutylicum* ATCC 824	One-step fermentation to accumulate butanol and butyryl-CoA. AAT from Strawberry and Apple were expressed in the host. Hexadecane was used as extractant	–	Strawberry SAAT yielded 50.07 mg/L and apple AAAT yielded 40.60 mg/L	Noh et al. (2018)
*C. beijerinckii* BGS1 and *C. tyrobutyricum* ATCC 25,755	Microbial co-culturing of two strains. lipase from *Candida* sp. were added	10 g/L butanol, 10 g/L butyrate	5.3 g/L	Cui et al. (2020)

## Production of BB by engineered *E. coli* with alcohol acyltransferases

In nature, when butyryl-CoA contacts butanol molecule in the presence of alcohol acyltransferases (AAT, EC 2.3.1.84), BB can be produced (Fig. 1c). AATs play a crucial role in ester biosynthesis by catalyzing the reaction between aliphatic and aromatic alcohols and the acyl-CoA molecules (Fig. 1c) (Gunther et al. 2011; Nancolas et al. 2017). AATs are different based on their specificities towards alcohol and acyl-CoA substrates. Therefore, some prefer short-chain substrates while others for example wax synthase/diacylglycerol acyltransferases (WS/DGATs) catalyze long-chain carbon substrates (Menendez-Bravo et al. 2017).

AATs are found in yeasts, filamentous fungi, bacteria, and many sweet fruits (Kruis et al. 2017), such as strawberry (Cumplido-Laso et al. 2012), banana (Beekwilder et al. 2004), melon (El-Sharkawy et al. 2005), mountain papaya (Balbontin et al. 2010), and apple (Defilippi et al. 2005). AATs from such fruits represent wide substrate specificities that range from C1 to C10 alcohols or even higher alcohols. AATs have also been known to contribute to ester biosynthesis in various microorganisms, for instance, *Saccharomyces cerevisiae* (Kruis et al. 2018; Saerens et al. 2010), *Yarrowia lipolytica* (Gao et al. 2018; Yu et al. 2020), *C*. *acetobutylicum* (Noh et al. 2018), and *E. coli* (Chacon et al. 2019). Another contribution of AAT on esters synthesis, particularly in *E. coli*, has been demonstrated by chloramphenicol *O*-acetyltransferases (CATs, EC 2.3.1.28), an AAT homolog isolated from *Streptococcus aureus* (Alonso-Gutierrez et al. 2013; Rodriguez et al. 2014). Very recently, by applying in silico mutagenesis with in vivo microbial screening assay on CATs from *Staphylococcus aureus*, a new candidate CAT namely CATsa was identified and showed the ability to work as a robust and efficient AAT (Seo et al. 2020). Apart from its ability to catalyze more acyl-CoA molecules and alcohols for numerous ester biosynthesis, it has more advantages in microbial ester biosynthesis than frequently used AATs originated from plants, simply because it originates from bacteria. To this enzyme, there are no upstream optimizations are needed before its expression in different hosts, for example, *E. coli* host strains.

Since AATs can be used to produce esters via microbial approach, *E. coli* has recently attracted increasing interest as an alternative microorganism for in vivo BB biosynthesis (Horton and Bennett 2006; Layton and Trinh 2014, 2016a, b). However, *E. coli* strains do not have native pathways for BB biosynthesis, which requires heterologous genetic modules, and pathways responsible for BB precursors (butyryl-CoA and/or butanol) production.

In 2014, Layton and Trinh (2014) used modularized ester pathways to biosynthesize BB in engineered *E. coli*. Here, butanol pathway from *C. acetobutylicum* was heterologously introduced into *E. coli* to generate butanol and butyryl-CoA, followed by expressing AAT from strawberry to convert expected precursors into BB. However, butanol could not be detected in the culture medium. In most cases, butanol, butyrate or other precursors were supplied, resulting in enhanced BB production. Using the above engineered *E. coli* (Layton and Trinh 2014), Layton and Trinh achieved BB of 36.8 mg/L, upon adding 2 g/L butanol. Two years later, the same research group used another strategy for BB production from carboxylate. In this case, *E. coli* was engineered by introducing heterologous “acid-to-ester submodular pathways,” so butyrate externally added to the culture medium could be converted into butyryl-CoA by acyl-CoA transferase. When the mutant strains were subjected to batch fermentation of glucose and butyrate, strains harboring AAT from *Fragaria ananassa* and *Fragaria vesca* could achieve 47.6 mg/L and 2.76 mg/L BB, respectively (Layton and Trinh 2016b).

Besides, in the presence of AATs, pure substrates can be supplemented as an alternative. For example, addition of 3 g/L butanol and 3 g/L 2-ketovalerate to a culture medium containing engineered *E. coli* strain expressed with AAT produced 14.9 mg/L BB (Rodriguez et al. 2014). In this strain, a branched-chain α-keto acid dehydrogenase complex converted 2-ketovalerate into butyryl-CoA, which later converted into BB in the presence of butanol and AAT from *S. cerevisiae*.

Efforts have also been made to introduce AATs into other host strains for BB production. For instance, when AAT from *Fragaria ananassa and Malus* sp. were, respectively, expressed in *C. acetobutylicum* (Noh et al. 2018), BB could be produced but the production metrics (titer and yield) were very low.

**Fig. 2:**
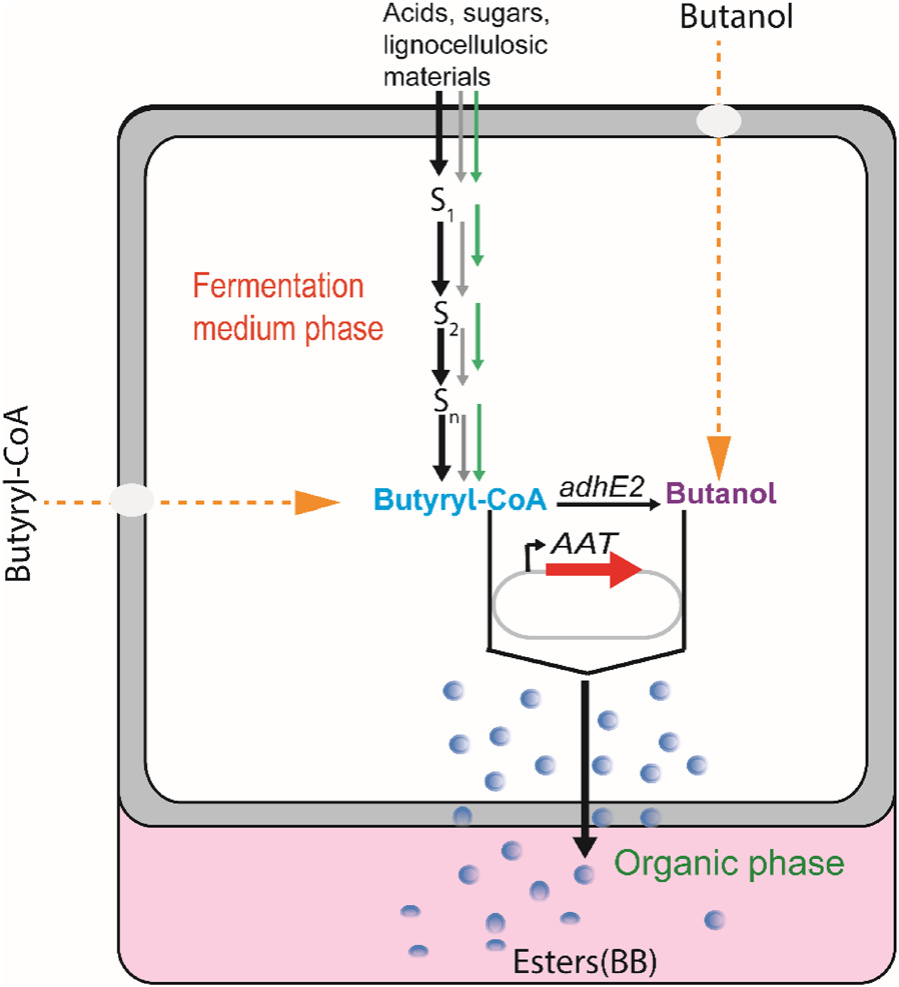
Combinatorial approaches attempted in single strain for BB biosynthesis. Black color denotes the native pathways responsible for butyryl-CoA and/or butanol accumulation through intracellular acyl-CoA molecules (in *Clostridia* species). The grey color shows the pathway optimizations by either overexpressing downstream acyl-CoA enzymes or introducing new enzymes with higher activity. Green color indicates the heterologous pathways aiming at improving the intracellular butyryl-CoA molecule for high butyrate and butanol availability (often in *E. coli*). In some cases, butyryl-CoA and/or butanol are supplemented in the fermentation system. The blue circles indicate BB produced. AAT modules should be introduced to catalyze the esterification reaction of butyryl-CoA with butanol into biosynthetic BB. *adhE2* (aldehyde/alcohol dehydrogenase)

Given that ester biosynthesis, particularly BB, remains a challenging process, numerous studies have linked this to the low activity of AAT. Besides, others argue that the low esters production metrics (titer, yield, and rate) are associated with either the regulation of esters precursors (butanol or butyryl-CoA) or enzyme (Fellman et al. 2000; Michael Knee and Hatfield 1981; Williams and Knee 1977). However all engineering approaches used to increase BB production (Fig. 2), particularly in an engineered single *E. coli* strain (Table 2) are far from to achieve high production. Thus, it is of paramount important to find an alternative approach to address this challenge.

**Table 2:** Recent advances on production of butyl butyrate by engineered *E. coli*

Host strains	Characteristics	Enzymes	Precursors supply	Titer	References
*E. coli* (Δ*adh*E, Δ*frd*, Δ*ldh*A, Δ*pta*, Δ*pfl*B, Δ*fnr*, Δ*yqh*D, Δ*adh*P, Δ*eut*G, Δ*yia*Y, Δ*yjg*B, Δ*fuc*O)	Batch culture of strain harboring KDHC to produce butyryl-CoA via 2-ketovalerate	EHTI	3 g/L butanol and 3 g/L 2-ketovalerate	14.9 mg/L	Rodriguez et al. (2014)
*E. coli* (Δ*adh*E, Δ*frd*, Δ*ldh*A, Δ*pta*, Δ*pfl*B, Δ*fnr*, Δ*yqh*D, Δ*adh*P, Δ*eut*G, Δ*yia*Y, Δ*yjg*B, Δ*fuc*O)	Batch culture of strain harboring KDHC to produce butyryl-CoA via 2-ketovalerate	CAT	3 g/L butanol and 3 g/L 2-ketovalerate	10.6 mg/L	Rodriguez et al. (2014)
*E. coli* (Δ*zwf*, Δndh, Δ*sfc*A, Δ*mae*B, Δ*ldh*A, Δ*frd*A, Δ*pox*B, Δ*pta*, Δ*fad*E)	Introduction of acyl-CoA and ethanol pathways (*ato*B, *hbd*, *crt*, *ter*, *pdc*, *adh*B), and 2-keto pathway	SAAT	Butyryl-CoA module from *Clostridium* pathway together with butanol module, and unstated quantity of butanol supplemented	36.83 mg/L	Layton and Trinh (2014)
*E. coli* (Δ*zwf*, Δndh, Δ*sfc*A, Δ*mae*B, Δ*ldh*A, Δ*frdA*, Δ*pox*B, Δ*pta*, Δ*fad*E)	Introducing acid-to-alcohol module (*pct, pdc, adh*B) followed by AAT module-to-ester	SAAT	2 g/L Butyrate	47.63 mg/L	Layton and Trinh (2016b)
*E. coli* (Δ*zwf*, Δndh, Δ*sfc*A, Δ*maeB*, Δ*ldh*A, Δ*frd*A, Δ*pox*B, Δ*pta*, Δ*fad*E)	Acid-to-ester submodule including acid-to-alcohol and isobutanol pathway (*als*S, *ilv*C, *ilv*D, *kiv*D, *adh*E, *pct*)	SAAT	2 g/L Butyrate	21.34 mg/L	Layton and Trinh (2016b)
*E. coli* expressing SAAT	Pure substrates undergone in vitro conversion using crude cell extracts	SAAT	1 g/L butanol and 3 g/L butyl-CoA	0.28 mg/L	Horton and Bennett (2006)
*E. coli* EB243–*E. coli EB243-*ΔadhE2::yciAh consortium	Introducing synthesis pathway genes (*ato*B, *hbd*, *crt*, *ter*, *adh*E2) and *fdh*, with deletions of a*dhE*, *eutE*, *yqhD*, *ackA*, *pta*, *hyc-hyp*, *fdhF*, *poxB*, *pck*, *fumB*, *fumAC*, *tdcD*, *mdh*, *focA*, *ppc*, *mgsA*, *yieP*, *stpA*, *yqeG*, and *yagM* for butanol and *yciAh* gene integrated into the chromosome of EB243*-*ΔadhE2	Lipase	–	7.2 g/L	Sinumvayo et al. (2021)

EHT1, alcohol o-acyltransferase from *S. cerevisiae*, CAT, chloramphenicol acetyltransferase, SAAT, alcohol acyltransferase from *Fragaria ananassa*

## Microbial consortium as a promising approach for BB biosynthesis

Recently, researches on microbial ester biosynthesis associate the low BB titer and yield with the limited availability of intrinsic precursors (butanol, butyryl-CoA and butyrate). This may be due simply to the multiple enzymatic steps (five for butyryl-CoA, six or seven for butyrate and seven for butanol) required for their in vivo synthesis. Since co-culture engineering has become an emerging approach to address concerns related to the bioproducts shortage, there is a need for separating the engineered strains comprising butyrate- and butanol-producing strains to build a microbial consortium for fermentation. Such consortium may facilitate the co-production of butanol and butyrate in the fermentation, hence improving BB biosynthesis.

## Microbial consortium constructed from different species

It has been reported that butanol may cause toxicity in *Clostridia* fermentation resulting in low product titers. Since butanol is known to be a substrate for BB production, solving the toxicity by carrying out esterification using butanol as one of the substrates during the fermentation would be appreciated. What is also interesting is that the BB produced is more hydrophobic than butanol, which makes it more likely easier to be extracted from the fermentation broth.

Based on this concept, Cui et al. (2020) used solventogenic *Clostridium* co-culture aiming to produce butanol and butyrate, which were subsequently converted by lipase into BB, then extracted by hydrophobic extractant. The consortium comprises two different species, *C. beijerinckii* BGS1 and *C. tyrobutyricum* ATCC25755, which could produce 6.8 g/L butanol and 9.7 g/L butyrate, respectively, during the co-culture fermentation process. Upon addition of lipase, 5.1 g/L of BB with a yield of 0.068 g/g glucose was produced. Here we recall that the more substrates increase in the consortium in the same or closer proportions, the greater the conversion rate into target ester increases. Therefore, in this consortium, it seems that the butanol produced by *C. beijerinckii* BGS1 is less than the butyrate concentration, which would be the reason accounting for low titer and yield of BB.

**Fig. 3:**
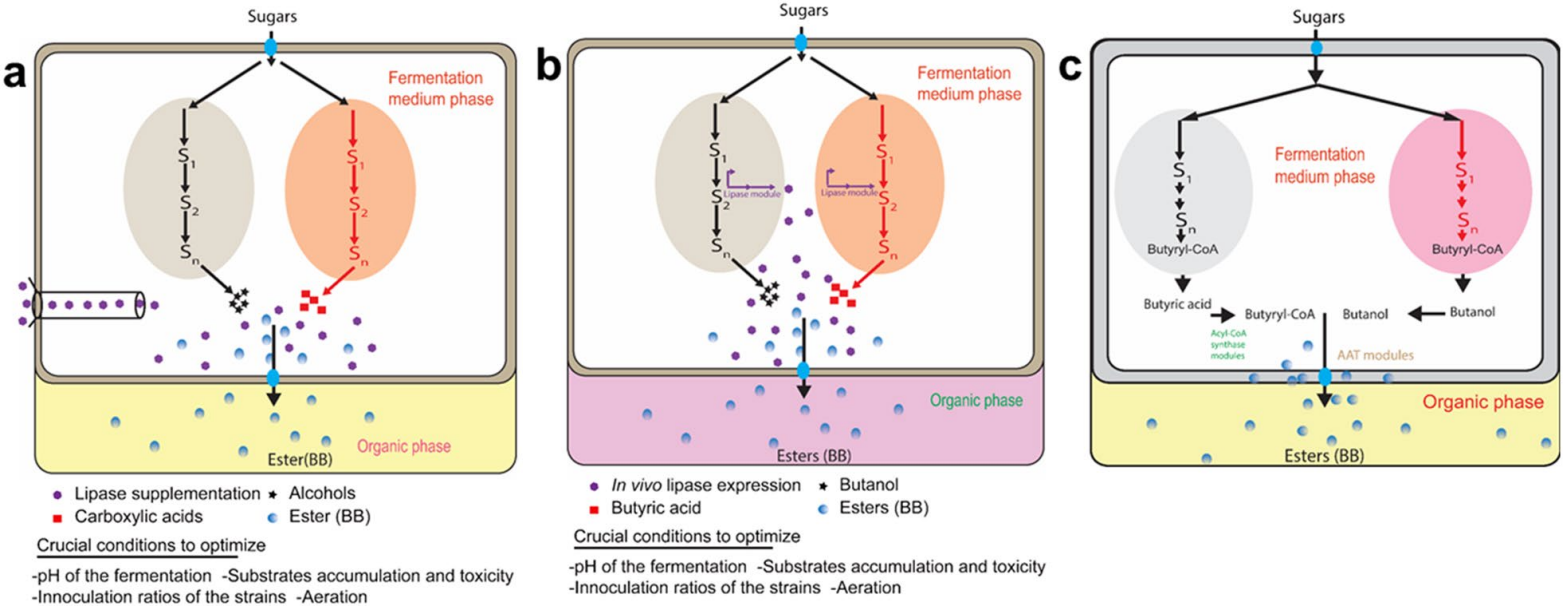
Schematic representation of possible microbial consortium approaches for efficient BB biosynthesis. Light grey color and light orange in (**a**) and (**b**) represent carboxylic acids- and alcohols-producing strains, respectively. The bright purple color (**a**) represents commercial lipase enzymes supplementation while in (**b**) stands for lipase heterologously expressed in one or both strains as an alternative to undergo esterification reaction. **c** Approach using a cognate consortium comprising butanol-producing strain and butyric acid-producing strain. Butyrate produced in the medium is converted by butyryl-CoA synthase into butyryl-CoA precursor. AAT represents alcohol acyltransferases involved in the last step to catalyze butyryl-CoA and butanol substrate into biosynthetic BB

In principle, the microbial consortium constructed using *Clostridia* strains should provide high yields because the process is performed under anaerobic conditions that do not require aeration and vigorous mixing. Therefore, the low titer and yield may be most likely due to the loss of carbon flux through the by-products accumulation such as isopropanol and acetone. To cope with this matter, metabolic engineering to remove competing pathways to maximize the selectivity of the target ester is needed. In short, while the different *Clostridia* species are employed to build a consortium, it is intricate if not impossible to synchronize their metabolism and the functionality of the consortium for a more balanced ratio of butanol/butyrate. Therefore, this opens the avenues for those who want to significantly increase ester biosynthesis using microbial consortium accompanied by optimizing key conditions as proposed in (Fig. 3a, b).

## A cognate consortium as a promising approach for BB production

As explained in the previous sections of this review, substrate shortage is considered as one of the most common problems for BB production when a single strain is engineered and used as a platform for ester biosynthesis. This approach may place a heavy burden on the strain, which leads to poor in vivo biosynthesis of BB precursors. Therefore, it requires supplementation of either external pure butanol or butyrate, or both at once for efficient BB synthesis. Nevertheless, this approach makes the process non cost-effective. The concept of microbial consortium engineering that recently became an emerging popular strategy for chemical biosynthesis can be used as an alternative to tackle these related challenges of shortage precursors. However, this is quite difficult for microbes from different species due to their incompatibility on the same growth medium which leads to inefficient microbial consortium applicability. Thus, the development and application of a cognate consortium is an imperative solution for efficient BB biosynthesis. A cognate consortium is explained by its partners, which belong to the same species and share almost the same metabolism pathway. In the cognate consortium, all partners similarly respond to the environmental stimuli (e.g., medium, temperature, pH, aeration, and product accumulation). In addition, a cognate concept favors the interaction of microbial partners in the consortium because all partners belong to the same species with similar behaviors. From these characteristics, a cognate consortium can manage the growth stability and prevents any conflict that may happen between consortium partners on sugar consumption. Since balancing the ratio of precursors in any consortium is one of the criteria to consider for efficient BB biosynthesis, application of a cognate consortium would be advisable simply because it may be easier to adjust the ratio of strain partners from the same species, which facilitates the control of the precursors accumulation than when strains are from different species.

Based on the abovementioned principle, Sinumvayo et al. (2021) developed a cognate microbial consortium for BB production. In this consortium, partner microbes independently produce butyrate and butanol for BB biosynthesis. Usually, a relatively equal amount of butyrate and butanol is a prerequisite at a similar rate to efficiently produce BB directly from glucose in the presence of a lipase. Therefore, building a microbial consortium to provide equimolar substrates should rely on the type of consortium partners and their inter-communication. In a cognate consortium constructed in this study, the first strain refers to a chromosomally engineered *E. coli* EB243 capable of efficiently producing butanol from glucose (Dong et al. 2017). The second strain was developed from EB243 by redirecting the carbon flow at the node of butyl-CoA, thus shifting the butanol production to butyrate production. The evolved butanol- and butyrate-producing *E. coli* strains were co-cultured and lipase was supplied to yield BB directly from glucose. This consortium is unique because both engineered *E. coli* strains share nearly the same metabolism except downstream of the butyryl-CoA. It demonstrates the scientific feasibility to researchers interested in microbial consortium not only for BB production but also for other esters that need a biotechnological production approach.

Consortium partners’ growth background is another key challenge that needs to be tackled for the high accumulation of precursors in the consortium. In the recent study conducted on BB production through microbial consortium (Sinumvayo et al. 2021), argued that construction and application of the cognate consortium is very important, especially when the target is to produce esters. Since most microbes do not possess complete pathways to generate precursors for esters synthesis, it is, therefore, necessary to systematically engineer the consortium partners based on the target precursor. However, metabolic change can also cause other factors perturbations, for instance, imbalanced cofactors regeneration, which may affect the performance of the strain resulting in low precursor accumulation. Besides, in a cognate consortium aiming to produce esters, no products produced should be the substrate of microbes in the consortium or toxic to the microbial communities in the consortium. Thus, for each attempt to construct a cognate consortium, great care and prudence should be taken on the abovementioned considerations.

While cognate consortium approach is a promising way to produce BB at the lab scale, it is still new for esters biosynthesis. So far, the yield and titer are higher than the yield when a non-cognate clostridia consortium is used. Presently, since the oil price is low, the biotechnological production of most chemicals is not economically competitive. To our opinion, a BB yield of higher than 0.4 g/g and a titer of higher than 40 g/L would be satisfactory. To achieve a yield of 0.4 g/g, we must conquer the incompatibility of the oxygen demand of the two strains in the consortium. To achieve a high titer, perhaps we need to consider fed-batch approach for adding more substrate, and to increase the volume of extractant so as the BB produced in the aqueous phase can be constantly removed.

## Conclusions and future perspectives

Butanol, butyrate, and butyryl-CoA are indispensable precursors for microbial BB biosynthesis. However, most microbes are unable to simultaneously biosynthesize these precursors. Therefore, biological combinatorial approaches comprising metabolic engineering and systematic screening of heterologous pathways have been proposed for precursors availability in a single strain (Fig. 2). It has been shown that producing two required precursors with an adequate concentration in a single platform strain for BB production is still an ongoing challenge. That is, without the addition of external precursors, BB cannot be produced to the desired extent. This approach is a problem as well since it increases the cost of the final BB produced.

Here, the microbial consortium concept may be considered as the solution for precursors supply to achieve a high BB titer. Xin et al. (2019) advised to develop a microbial consortium comprising clostridia species and the use of indigenous lipase in the lieu of supplementation. However, the fact that the mutually beneficial interactions are missing among strains from different species, it is concerning to achieve stable and reproducible ratios as well as their performance in such consortium. For example, if one strain grows at aerobic conditions while the other prefers anaerobic, it will be difficult if not impossible to match such fermentation conditions in a well-mixed bioreactor for efficient BB production. In this review, we have highlighted the importance of using a “cognate” approach rather than a non-cognate strategy (using a single strain or a consortium comprising strains from different species).

Therefore, future works on BB production are advised to use a cognate consortium concept, as the potential approach to efficiently produce BB. To achieve this, it is highly recommended to construct consortium partners with similar background characteristics at the forefront. Here, by using engineered *E. coli* strains from recently published works we propose a new cognate consortium which my enhance BB production. In the first study, Zhao et al. (2019) developed an engineered *E. coli* able to utilize xylose as a carbon source. This strain can tolerate oxygenation hence contributing to butanol production. In the second study glucose-utilizing engineered *E. coli* strain was constructed and showed a promising butyrate titer and yield under aeration conditions (Sinumvayo et al. 2021). These two-engineered *E. coli* strains are claimed to have nearly identical genomic backgrounds, which may makes them good candidates for constructing a cognate consortium for efficient BB production from glucose and xylose that can be provided by using cellulosic biomass hydrolysates. This cognate consortium with a shape in two different “heads” (using xylose or glucose) but the same “body” (from glycolysis to butyryl-CoA then diverging in two routes, for butyrate and butanol) would be appreciated for future BB biosynthesis. Despite this interest, no one as far as we know has attempted to construct such a cognate microbial consortium from mixed sugars (C5 and C6). Thus, this idea is a new proposal not only limited to BB production, but which can go beyond limits and be applied to many other esters in the future.

In addition, as reviewed recently (Xin et al. 2019), it is arguable that the shortage of butyryl-CoA in a single strain may be another problem for efficient BB biosynthesis. This is because in a single strain a part of butyryl-CoA is converted into butanol while high butyryl-CoA and butanol are needed for high BB biosynthesis. Therefore, the design and development of a microbial cognate consortium strategy (Fig. 3c) to refill butyryl-CoA partially used for butanol production is highly advised for increasing BB biosynthesis. In short, the cognate consortium concept discussed in this review will address the most common problem in BB biosynthesis especially concerning substrates supplementation, which costs the fermentation process. Since partners in a cognate consortium are of the same species, the concept will solve the growth challenge frequently observed in the normal microbial consortium often results in poor performance. This concept may not be limited to BB biosynthesis, but it can go beyond the limit and be applied to many other future studies interested in esters biosynthesis.

To date, findings emphasize that lipases are the most used enzymes to catalyze the esterification of butyrate with butanol for efficient BB synthesis. This is largely due to their broad substrate specificity, enantio-selectivity, stability in organic solvents as well as at extreme temperatures and pHs compared to other types of enzymes. However, the problem remains at their cost since an excellent balance between the output (titer, yield)/inputs (precursors, enzymes) is needed. To overcome constraint related to cost, overproducing recombinant lipases for selective BB biosynthesis should be considered with in-depth study of lipase engineering since each lipase producing species has its own set of lipases with different levels of activity, stability, and substrate selectivity, fitting its physiological and metabolic requirements (Contesini et al. 2020).

It is known that the esterification reaction leaves water in the system, which therefore hydrolyzes the formed esters to their original substrates. This problem is most evident when ester synthesis is performed in situ or in vivo fermentation. To avoid this problem, ester formation must occur simultaneously with the recovery process from the fermentation mixture. Zhang et al. indicated that the partition coefficient of ester was found to be about 10^4^ times higher than that of its substrates, this indicates that BB is much more hydrophobic compared to butanol and butyrate in the fermentation broth and can be easily extracted into the organic solvent phase. The organic solvent containing BB will be subjected to distillation to obtain purified BB, if operated on an industrial scale. During the fermentation, the appropriate organic extractant, e.g., hexadecane, can be added to the fermentation then BB produced is extracted from the medium. Here the organic solvent and the fermentation broth should be thoroughly mixed to speed up the condensation of substrates and the extraction of BB at once. However, some extractants inhibit cell growth and severely impede the smooth process of fermentation. A recent study aimed at producing BB in *C. acetobutylicum* was able to investigate the effect of some extractants on cell growth and esters recovery capacity and found that hexadecane has a high affinity for BB and has no negative effect on cell growth and fermentation performance (Zhang et al. 2017). If operated on an industrial scale, the hexadecane phase containing BB can be subjected to distillation to obtain the purified BB, and reused in the extractive fermentation.

## Data Availability

Not applicable.

## Abbreviations

BB: Butyl butyrate
CoA: Coenzyme A
S1: Glucose
S2: Acetyl-CoA
Sn: Acetoacetyl-CoA
LCS: Recombinant lipase from *Candida* sp., expressed in *Aspergillus** niger*; Novozymes Lipozyme^®^ CALB
NADH: Nicotinamide adenine dinucleotide
AAT: Alcohol acyltransferase
